# Estimated Incidence of Symptomatic Lyme Borreliosis Cases in Lublin, Poland in 2021

**DOI:** 10.3390/microorganisms11102481

**Published:** 2023-10-03

**Authors:** Emily Colby, Julia Olsen, Frederick J. Angulo, Patrick Kelly, Kate Halsby, Andreas Pilz, Urszula Sot, Tomasz Chmielewski, Katarzyna Pancer, Jennifer C. Moïsi, Luis Jodar, James H. Stark

**Affiliations:** 1 Vaccines, Antivirals, and Evidence Generation, Pfizer Biopharma Group, Collegeville, PA 19426, USA; 2 Pfizer Vaccines, Tadworth, Surrey KT20 7NS, UK; 3 Vaccines, Pfizer Corporation Austria, 1210 Vienna, Austria; 4 Vaccine Medical Affairs, Pfizer Poland Inc., 02-092 Warsaw, Poland; 5 National Institute of Public Health, 00-791 Warsaw, Poland; 6 Pfizer Vaccines, 75014 Paris, France; 7 Vaccines, Antivirals, and Evidence Generation, Pfizer Biopharma Group, Cambridge, MA 02139, USA

**Keywords:** Lyme borreliosis, tick-borne disease, surveillance, seroprevalence, incidence, Poland

## Abstract

Lyme borreliosis (LB), the most common tick-borne disease in Europe, is endemic to Poland. Despite public health surveillance with mandatory reporting of LB cases by physicians and laboratories, many symptomatic LB cases are not included in surveillance in Poland. We estimated the extent of the under-ascertainment of symptomatic LB cases via surveillance in the Polish province of Lublin to better understand Poland’s LB burden. The number of incident symptomatic LB cases in Lublin in 2010 was estimated from two seroprevalence studies conducted among adults in Lublin, as well as estimates of the proportion of asymptomatic LB cases and the duration of LB antibody persistence. The estimated number of incident symptomatic LB cases was compared to the number of surveillance-reported cases in Lublin to derive an under-ascertainment multiplier. This multiplier was applied to the number of surveillance-reported cases in 2021 to estimate the number and population-based incidence of symptomatic LB cases in Lublin in 2021. We estimate that there are 5.9 symptomatic LB cases for every surveillance-reported LB case in Lublin. Adjusting for under-ascertainment, the estimated number of symptomatic LB cases in Lublin in 2021 was 6204 (population-based incidence: 467.6/100,000). After adjustment for under-ascertainment, the incidence of symptomatic LB in Lublin, Poland, is high.

## 1. Introduction

Lyme borreliosis (LB), caused by the spirochete *Borrelia burgdorferi* (*B. burgdorferi*) sensu lato (s.l.), is the most common tick-borne disease in Europe [1]. Clinical manifestations of LB include erythema migrans and several forms of disseminated disease, such as Lyme neuroborreliosis and Lyme arthritis [2]. LB is endemic in Western, Eastern, and Northern Europe [3]. In Poland, LB cases that are identified by clinicians and laboratories are reported to the National Institute of Public Health as part of mandatory public health surveillance. Nationwide surveillance-reported LB incidence in Poland increased by 39% from 23.6/100,000 population in 2010 to 32.8/100,000 population in 2021 (nationwide population: 38.3 million in 2021) [4].

**Figure 1:**
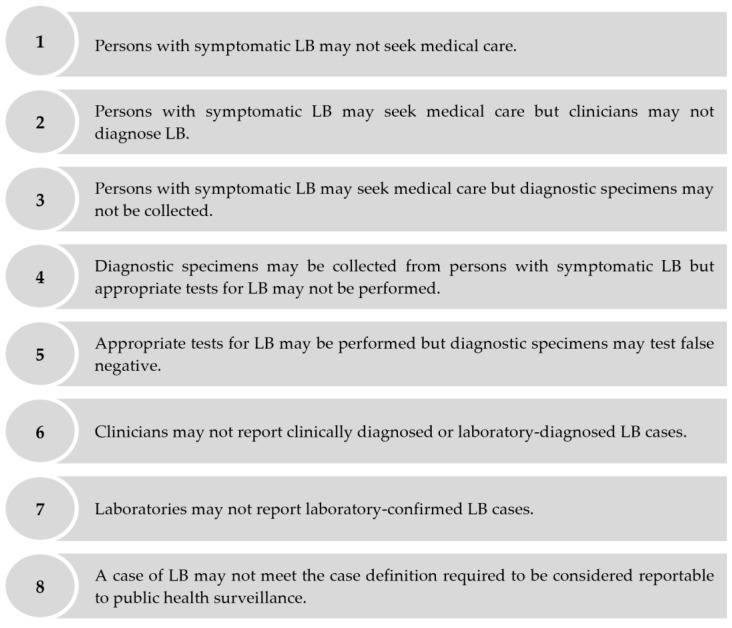
Reasons why public health surveillance may fail to ascertain a person with symptomatic LB who seeks medical care as an LB case.

Despite mandatory reporting to public health surveillance, symptomatic cases of LB may be under-ascertained for several reasons including, but not limited to, the following: persons with symptomatic LB may not seek medical care; patients with symptomatic LB may seek medical care, but clinicians may not diagnose LB; patients with symptomatic LB may seek medical care, but clinicians may not report clinically diagnosed or laboratory-diagnosed LB cases, etc. (Figure 1). Several approaches estimate the extent of the under-ascertainment of symptomatic cases via public health surveillance through an under-ascertainment “multiplier.” Under-ascertainment multipliers can be applied to surveillance-reported incidence to derive adjusted estimates of population-based incidence [5,6]. One approach to estimate under-ascertainment multipliers is to compare the estimated incidence of symptomatic infections derived from seroprevalence studies to the number of surveillance-reported cases [7,8,9]. We used this seroprevalence-based approach to estimate the extent of the under-ascertainment of symptomatic LB cases in public health surveillance to better understand the burden of LB in the Polish province of Lublin. We focused on symptomatic cases only, as persons with asymptomatic LB are not expected to seek medical care and, therefore, cannot be ascertained via public health surveillance [10].

A seroprevalence-based approach for estimating under-ascertainment multipliers for symptomatic LB has previously been applied in Finland and Germany [11,12]. We now expand this analysis to Poland, where the LB disease burden remains poorly understood, and epidemiologic burden of disease data are sparse [13]. This study contributes to the existing literature because it sheds light on variations in LB endemicity across European countries.

## 2. Materials and Methods

### 2.1. Seroprevalence Literature Search

A systematic literature review of seroprevalence studies conducted in Poland and published from 2005 to 2020, described elsewhere [14], failed to identify any nationwide seroprevalence studies in Poland. However, two representative seroprevalence studies conducted among adults in Lublin, one of the 16 provinces in Poland (Lublin adult population: 1.3 million in 2021), were identified [15,16].

The first seroprevalence study was conducted using specimens collected from 1998 to 2007 from 144 participants, 94 of whom were rural inhabitants engaged in farming (mean age: 56.3 ± 14.3 years) and located 11 km from the Lublin city border. The remaining 50 participants made up a comparison group of healthy Polish adult blood donors (mean age: 29.7 ± 5 years) living in the city of Lublin [15]. As rural farmers have a higher risk of LB infection than the general population, we excluded the 94 participants engaged in farming and only included the study’s comparison group of 50 participants in our analysis to better represent the risk of LB infection in a general population. This study tested blood samples with an enzyme-linked immune assay (ELISA) (*Borrelia* recombinant IgM and *Borrelia* recombinant IgG, Bellco Biomedica GmbH, Vienna, Austria) and a confirmatory line blot test (*Borrelia* recom Line IgM and *Borrelia* recom Line IgG, Mikrogen, Neuried, Germany).

The second seroprevalence study was conducted using specimens collected in 2013 from 320 individuals, 275 of whom were forestry workers and farmers (mean age: 44 ± 11 years) in the provinces of Lublin and Podlaskie. The remaining 45 participants comprised a comparison group of 45 Polish adults (mean age: 27 ± 9 years) living in Lublin with no occupational exposure to ticks [16]. As with the first seroprevalence study, we only included this study’s comparison group of 45 participants in our analysis to better represent the risk of LB infection in a general population. This study tested blood samples with an ELISA (Euroimmun, Lübeck, Germany; specific test kit not specified) to assess the presence of IgM/IgG antibodies and a confirmatory western blot test (Mikrogen, Neuried, Germany; specific test kit not specified) to determine the presence of IgM/IgG antibodies.

According to the Centers for Disease Control and Prevention (CDC), a two-tier methodology should be used in the serologic testing of Lyme disease [17]. As per CDC recommendations, this methodology should consist of a sensitive enzyme immunoassay (EIA) or immunofluorescence assay as a first-tier test, followed by a western immunoblot assay (or a second EIA with sensitivity and specificity that is equal to or higher than a western immunoblot assay) as a second-tier test for specimens yielding equivocal or positive results. In both studies, a two-tier testing protocol, comparable to CDC recommendations, was used; these two studies reported the seroprevalence of specimens that tested positive via a confirmatory second-tier test following a first-tier ELISA test.

### 2.2. Additional Literature Searches

Two additional PubMed literature searches, described elsewhere [11,12], were conducted to estimate two parameters that were needed to derive under-ascertainment multipliers. The first search identified studies that estimate the duration of antibody detection in persons infected with *B. burgdorferi* s.l. [18,19,20,21,22]. The second search identified studies that estimate the proportion of persons infected with *B. burgdorferi* s.l. who are asymptomatic [23,24,25,26]. None of the articles identified in these two searches included data from Poland. Therefore, a third PubMed search was performed for articles that reported the *B. burgdorferi* s.l. genospecies distribution among isolates from Poland. Three such articles were found [27,28,29]. In considering the three genospecies articles from Poland in combination with the four articles that were identified to estimate the asymptomatic proportion, which were conducted in Sweden, Finland, Austria, and the Netherlands, *B. afzelii* and *B. garinii* were the most common genospecies (Table 1).

### 2.3. Estimation of the Number and Incidence of Adult Symptomatic LB Cases in Lublin in 2021

**Figure 2:**
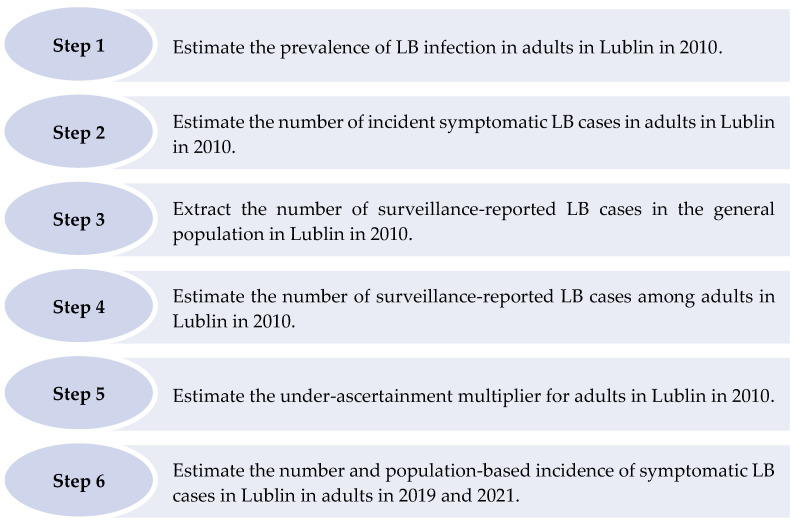
Steps taken to estimate the number and population-based incidence of symptomatic Lyme borreliosis cases in Lublin in adults in 2019 and 2021.

The six steps described below were taken to derive under-ascertainment multipliers and estimate the number and incidence of symptomatic LB cases among adults in Lublin (Figure 2).

#### 2.3.1. Step 1: Estimate the Prevalence of LB Infection in Adults in Lublin in 2010

The results from the two seroprevalence studies were analyzed together to estimate the prevalence of LB infection in Lublin adults [15,16]. We assumed this combined result to reflect the seroprevalence for adults in 2010, the midpoint between the last years of sample collection of the two seroprevalence studies.

#### 2.3.2. Step 2: Estimate the Number of Incident Symptomatic LB Cases in Adults in Lublin in 2010

We estimated ten years of IgG antibody detection based on the median duration from the studies that were identified in our first literature search [18,19,20,21,22]. We also estimated that 50% of infected persons are asymptomatic based on the median proportion from the studies found in our second literature search [23,24,25,26]. The estimated number of incident adult symptomatic LB cases in 2010 was derived using the formula below:

$$Estimated\;number\;of\;incident\;adult\;symptomatic\;LB\;cases\;in\;Lublin=\frac{\mathrm{Seroprevalence}\;*\mathrm{Population}\;\mathrm{size}\;*\;50\%\;\mathrm{Asymptomatic}\;\mathrm{infections}}{\mathrm{Ten}-\mathrm{year}\;\mathrm{duration}\;\mathrm{of}\;\mathrm{antibody}\;\mathrm{detection}}$$

#### 2.3.3. Step 3: Extract the Number of Surveillance-Reported LB Cases in the General Population in Lublin in 2010

Public health surveillance data were retrieved from the National Institute of Public Health in Poland [30].

#### 2.3.4. Step 4: Estimate the Number of Surveillance-Reported LB Cases among Adults in Lublin in 2010

The two seroprevalence studies that were used to estimate the prevalence of LB infection in Lublin in 2010 were conducted among adult populations aged 18–36 years. However, age-based LB surveillance data were only available for the nationwide level for the years 2015 to 2019. All other LB surveillance data from Poland only reported the total number of cases without specifying ages. Therefore, we multiplied the total number of surveillance-reported cases in Lublin in 2010 by the percentage of surveillance-reported cases in adults (aged 20 years and older) nationwide from 2015 to 2019 to estimate the number of adult surveillance-reported cases in Lublin in 2010.

#### 2.3.5. Step 5: Estimate the Under-Ascertainment Multiplier for Adults in Lublin in 2010

The estimated number of adult incident symptomatic cases in Lublin in 2010 was compared to the estimated number of adult surveillance-reported cases in Lublin in 2010 to derive an under-ascertainment multiplier.

#### 2.3.6. Step 6: Estimate the Number and Population-Based Incidence of Symptomatic LB Cases in Adults in Lublin in 2021 (and 2019)

The under-ascertainment multiplier was applied to the estimated number of surveillance-reported cases in Lublin adults in 2019 and 2021, once again estimated from the percentage of surveillance-reported cases in adults (aged 20 years and older) nationwide from 2015 to 2019. The surveillance data were retrieved from the National Institute of Public Health [30]. This step was conducted for LB surveillance data from 2021 because, at the time of this study, 2021 was the most recent year with published LB surveillance data available. Step 6 was also conducted for LB surveillance data from 2019 because of a notable decline in reported LB cases beginning in 2020. The adult population data were obtained from Poland’s statistics bureau, Statistics Poland [31].

### 2.4. Duration of Antibody Detection Sensitivity Analysis

A sensitivity analysis was conducted to evaluate the impact of using five- and twenty-year durations of antibody detection to derive under-ascertainment multipliers and estimates of the number and population-based incidence of adult symptomatic LB cases in Lublin in 2021.

## 3. Results

### 3.1. Step 1: Estimate the Prevalence of LB Infection in Adults in Lublin in 2010

**Figure 3:**
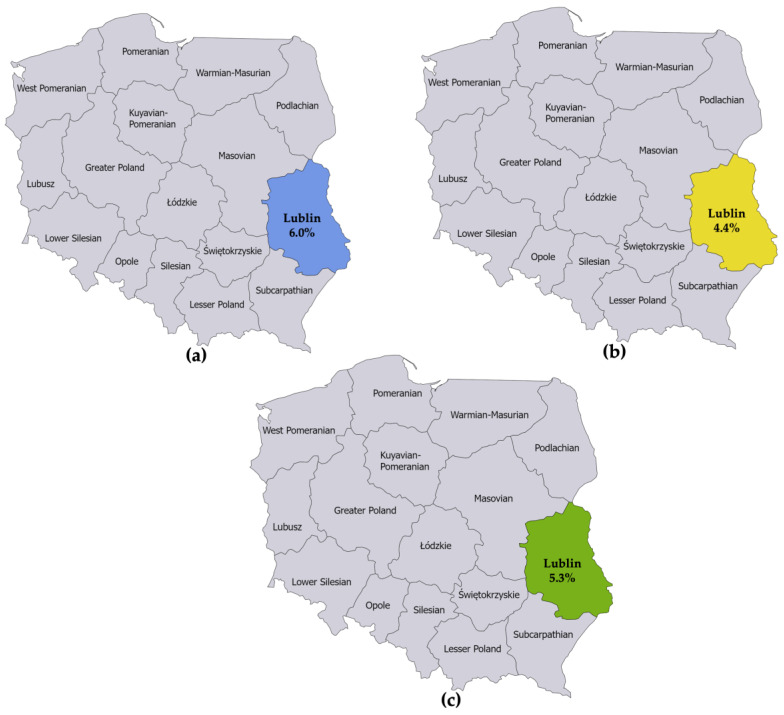
(**a**) Seroprevalence of IgG and IgM antibodies against *B. burgdorferi* sensu lato in specimens collected from 1998 to 2007 from 50 healthy adult blood donors living in the province of Lublin; (**b**) Seroprevalence of IgG and IgM antibodies against *B. burgdorferi* sensu lato in specimens collected in 2013 from 45 healthy adults not occupationally exposed to ticks living in the province of Lublin; (**c**) Estimated seroprevalence of IgG and IgM antibodies against *B. burgdorferi* sensu lato in the province of Lublin derived from combining the estimates from the studies referenced in (**a**,**b**).

The first seroprevalence study reported an LB prevalence of 6.0% among adults in Lublin [15]. The second seroprevalence study reported an LB prevalence of 4.4% among adults in Lublin [16]. The numerators and denominators were summed to derive a single seroprevalence estimate for Lublin. This resulted in an LB prevalence estimate of 5.3% for adults in Lublin in 2010 (Figure 3).

### 3.2. Step 2: Estimate the Number of Incident Symptomatic LB Cases in Adults in Lublin in 2010

In considering the prevalence of LB infection that was estimated from the seroprevalence data with the estimates of the proportion of asymptomatic cases and the duration of antibody detection, we estimated that there were 3975 incident LB cases among adults in Lublin in 2010 (Table 2).

### 3.3. Step 3: Extract the Number of Surveillance-Reported LB Cases in the General Population in Lublin

**Figure 4:**
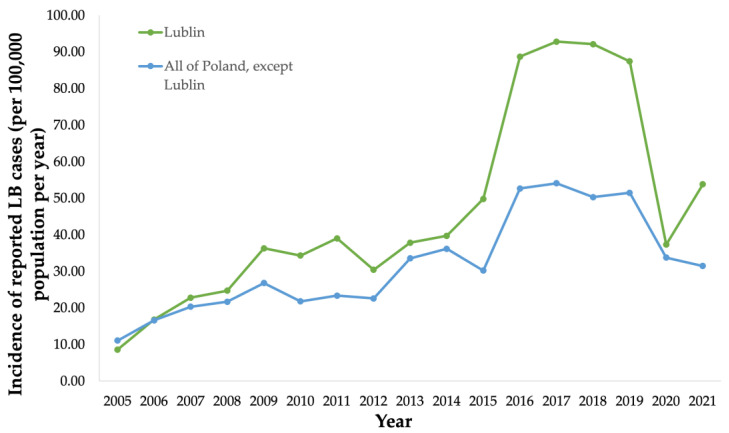
Incidence (per 100,000 population) of surveillance-reported Lyme borreliosis (LB) cases in the province of Lublin from 2005 to 2021, and incidence (per 100,000 population) of surveillance-reported LB cases in all provinces in Poland except Lublin from 2005 to 2021.

Public health surveillance data were collected from the National Institute of Public Health (NIH) in Poland from 2005 to 2021 (Figure 4). Public health surveillance reported 739 LB cases (34.3/100,000 population) in Lublin in 2010 [30] (Figure 4).

### 3.4. Step 4: Estimate the Number of Surveillance-Reported LB Cases among Adults in Lublin in 2010

From 2015 to 2019, 91% of surveillance-reported LB cases nationwide in Poland were among the adult population (aged 20 years and older). We thus estimated 672 surveillance-reported cases among adults in Lublin in 2010 (91% of 739 surveillance-reported cases among the total population).

### 3.5. Step 5: Estimate the Under-Ascertainment Multiplier for Adults in Lublin in 2010

The estimated number of incident symptomatic cases among adults in 2010, relative to the estimated number of adult surveillance-reported cases in 2010, yielded an under-ascertainment multiplier of 5.9 symptomatic LB cases for every surveillance-reported LB case in Lublin (Table 2).

### 3.6. Step 6: Estimate the Number and Incidence of Symptomatic LB Cases in Adults in Lublin in 2021 (and 2019)

Public health surveillance reported 1843 and 1122 LB cases among the total population in Lublin in 2019 and 2021, respectively. We again estimated that 91% of these cases were from adults, resulting in an estimated 1677 (119.3/100,000 adult population) and 1021 (76.9/100,000 adult population) surveillance-reported LB cases for adults in Lublin in 2019 and 2021, respectively. Applying the under-ascertainment multiplier to this surveillance data yielded estimates of 9,894 symptomatic LB cases (703.6/100,000 population) among adults in Lublin in 2019 and 6204 symptomatic LB cases (467.6/100,000 population) among adults in Lublin in 2021 (Table 2).

### 3.7. Duration of Antibody Detection Sensitivity Analysis

Using a five-year antibody detection duration, the under-ascertainment multiplier estimated for adults in Lublin was 11.8. Applying this under-ascertainment multiplier to the estimated number of surveillance-reported LB cases among adults in Lublin in 2021 resulted in 12,048 symptomatic adult LB cases (908/100,000 population) (Table 3). Using a twenty-year duration of antibody detection, the under-ascertainment multiplier was 3.0, yielding an estimated 3063 symptomatic adult LB cases (230.8/100,000) in Lublin in 2021 (Table 3).

## 4. Discussion

Poland conducts LB public health surveillance via mandatory reporting by clinicians and laboratories in all sixteen provinces. Nevertheless, using seroprevalence data from the province of Lublin, we estimate that there are approximately six symptomatic adult LB cases for every surveillance-reported adult LB case in Lublin. After adjusting for under-ascertainment, the population-based incidence of symptomatic LB was 467.6/100,000 adult population in Lublin in 2021. This adjusted incidence is remarkably higher than the incidence we estimate was reported through public health surveillance for adults in Lublin in 2021 (76.9/100,000 adult population) [3,32].

We do not know the extent to which Lublin is representative of all of Poland. Only approximately 6% of the Polish adult population resides in Lublin, and 9% of surveillance-reported LB cases per year in Poland were from Lublin in 2019 and 2021. If we apply the derived multiplier of 5.9 to the nationwide LB surveillance data estimated for adults (91% of 12,500 total reported cases), the estimated number of incident symptomatic cases in adults in 2021 is 67,113, and the estimated population-based symptomatic LB incidence is 176.2/100,000 adult population. However, we do not know if the derived multiplier is representative of all of Poland or if the multiplier in other provinces in Poland would be higher or lower than the multiplier we estimated. Furthermore, the extent of the under-ascertainment of symptomatic LB cases via public health surveillance may vary across provinces, regions, and countries due to several factors, including care-seeking behaviors, a clinical awareness of LB, the sensitivity and specificity of diagnostic testing, and the completeness of disease reporting.

Other studies have suggested that cases of LB are under-ascertained by the public health surveillance system in European countries such as Poland [4,33]. However, to our knowledge, this study is the first to estimate the extent of LB under-ascertainment within a province of Poland. We acknowledge that this seroprevalence-based approach of deriving multipliers to estimate the under-ascertainment of LB via surveillance has previously been used in Northern Europe in Finland and Western Europe in Germany [11,12]. However, deriving such multipliers in Lublin, Poland adds novel insight to the existing LB burden of disease literature by revealing how the under-ascertainment of symptomatic LB varies across Northern, Western, and now Central Europe (i.e., Finland, Germany, and Poland, respectively).

In Finland, there were an estimated three symptomatic LB cases for every surveillance-reported LB case among adults [11], compared to twelve among adults and nine among children in the nine federal states that conduct public health surveillance for LB in Germany [12]. The estimated incidence of symptomatic LB in Finland was 520/100,000 in 2021 (compared to 153 surveillance-reported cases/100,000), and the estimated incidence of symptomatic LB in Germany was 408/100,000 in 2021 (compared with 31 surveillance-reported cases/100,000).

The quality of the estimates of under-ascertainment multipliers derived from a seroprevalence-based approach depends on the availability of high-quality seroprevalence and public health surveillance data. We used two published seroprevalence studies conducted in Lublin that used a standard two-tier laboratory testing approach to estimate the proportion of the population infected with *B. burgdorferi* s.l. in Lublin. One additional, high-quality seroprevalence study that was conducted in Lublin found a seroprevalence estimate of 12.5% [34]. However, this study was excluded from our analysis as it did not utilize a two-tier testing approach, which may cause the seroprevalence to be overestimated. Although the two seroprevalence studies used in our analysis employed an appropriate laboratory testing approach, these studies had several limitations. Both studies had small sample sizes of 45 and 50 adults. Given the similar and small sample sizes of the two seroprevalence studies, we combined the numerators and denominators by adding them, rather than using weighting, to obtain a single seroprevalence estimate. However, this approach may still contain some error. Future Poland-based LB seroprevalence studies with more extensive participation from the general population and which report LB prevalence stratified by age would provide more robust estimates. Such studies would also allow for more complex methods that evaluate how the incidence of LB infection varies with age [35]. Research and surveillance data suggest that LB incidence fluctuates based on age, with incidence being the most elevated in children and older adults [36]. However, our study could not easily account for such age-based variation given the lack of high-quality age-stratified LB seroprevalence data available for Poland. The quality of the public health surveillance system for LB in Poland is high, with the mandatory notification of LB cases by clinicians and laboratories to the National Institute of Public Health. The quality and availability of this surveillance data lend credibility to our under-ascertainment multiplier estimates.

The validity of the seroprevalence-derived under-ascertainment multiplier estimate depends on the accuracy of several assumptions that were estimated from a limited number of available studies. Based on the published literature, the percentage of infected persons who were asymptomatic was estimated to be 50%. If a higher proportion of infected cases are asymptomatic, the estimated number of symptomatic cases would be lower, and vice versa. Further, we assumed a ten-year duration of IgG antibody detection. With a shorter duration of antibody detection, the estimated number of symptomatic cases and under-ascertainment multipliers would be higher, and vice versa. The assumptions listed above contain a level of uncertainty that is difficult to account for in our analyses. As a result, we cannot calculate meaningful confidence intervals around our reported under-ascertainment multipliers; attempting to do so may convey a false measure of precision. For this reason, we have excluded the use of confidence intervals from our analysis.

Our study has several limitations. First, the two seroprevalence studies that were used to derive the under-ascertainment multipliers were conducted in adult populations. However, LB surveillance data were only available in aggregate for adults and children for 2010, the year that we used to derive an under-ascertainment multiplier. Thus, we estimated the number of reported cases among adults in 2010 using nationwide age-specific surveillance data from the years for which data were available (2015–2019) to derive the under-ascertainment multiplier. Second, the comparison groups from the two seroprevalence studies that were used in our analysis had small sample sizes of 45 and 50 adults. When combined, the overall proportion of LB seroprevalence from these two studies resulted in five positive specimens out of ninety-five total specimens. By combining these two studies’ results, we account for some of the potential instability in our estimates due to the small sample sizes. However, future studies with larger sample sizes from the general population would produce more stable results. Third, there was a remarkable decline in the incidence of surveillance-reported LB cases from 2019 to 2021. The reasons for this decline are unclear. However, studies have posited that the SARS-CoV-2 pandemic may have impacted the LB surveillance reporting that was observed in Poland in 2020 and 2021 [37]. Because of the decline in incidence from 2019 to 2021, we estimated the under-ascertainment-adjusted symptomatic LB incidence for 2019 and 2021 separately. Fourth, public health surveillance may have changed over time, becoming more or less sensitive due to changes in the clinical awareness of disease, the likelihood of laboratory testing, or the sensitivity of the laboratory tests used, so under-ascertainment multipliers from 2010 may not be applicable in 2021. Fifth, the validity of our results is limited due to the lack of age-stratified LB seroprevalence and surveillance data.

## 5. Conclusions

We estimated the extent of the under-ascertainment of symptomatic LB via public health surveillance and identified a high incidence of symptomatic LB among adults in Lublin. A seroprevalence-based approach for estimating the incidence of symptomatic LB cases could be applied in additional countries that conduct surveillance for LB and have representative LB seroprevalence data available; this would likely demonstrate a higher incidence of symptomatic LB than is currently recognized. Given the high burden of LB, public health efforts, such as the development of a safe and efficacious vaccine, are needed to enhance disease prevention and control [38].

## Figures and Tables

**Table 1:** Genospecies distribution in the articles with data from Poland and the articles used to estimate the proportion of asymptomatic infections.

Articles with Data from Poland ^1^	*B. afzelii*	*B. garinii*	*B. burgdorferi* s.s.	*B. valaisiana* ^2^	*B. spielmenii* ^3^	*B.miyamotoi* ^2^	*Untypeable*
Grygorczuk et al., 2013 [27] (n = 30 sera samples)	83.3%	10.0%	6.7%	--	--	--	--
Kubiak et al., 2012 [28] (n = 259 sera samples)	29.2%	15.8%	30.4%	--	4.3%	--	20.0%
Wojciechowska-Koszko et al., 2021 [29] (n = 53 sera samples)	32.0%	60.0%	--	--	--	--	8.0%
Articles used to estimate the proportion of infections that are asymptomatic							
Hofhuis et al., 2013 [23] (n = 56 *Ixodes ricinus* ticks removed from tick-bitten persons	64.3%	19.6%	12.5%	7.1%	--	--	--
Markowicz et al., 2021 [24] (n = 194 *Ixodes ricinus* ticks removed from tick-bitten persons)	68.0%	16.5%	7.7%	7.2%	--	--	--
Wilhelmsson et al., 2013 ^4^, [25] Wilhelmsson et al., 2016 ^4^ [26] (n = 178 *Ixodes ricinus* ticks removed from tick-bitten persons)	48.3%	23.6%	2.2%	7.9%	3.0%	1.0%	13.0%

^1^ Results shown refer only to sera that tested positive for IgG antibodies against *B. burgdorferi* sensu lato. ^2^ None of the articles with data from Poland and only one article that was used to estimate the proportion of asymptomatic infections (Wilhelmsson et al., 2013 [25]) reported information for *B. valaisiana* and *B. miyamotoi* genospecies. ^3^ Only one article with data from Poland (Kubiak et al., 2012 [28]) and one article that was used to estimate the proportion of asymptomatic infections (Wilhelmsson et al., 2013 [25]) reported information for *B. spielmenii*. ^4^ Genospecies distribution and number of ticks tested is derived from the combination of both studies.

**Table 2:** Under-ascertainment multipliers in Lublin in 2010 and estimated number of symptomatic Lyme borreliosis (LB) cases in Lublin adults, 2019 and 2021.

Estimated Number of Surveillance-Reported LB Cases in Adults, 2010	Estimated Number of Incident Symptomatic LB Cases in Adults, 2010	Under-Ascertainment Multiplier(Based on 2010 Data)	Estimated Number of Surveillance-Reported LB Cases in Adults, 2019	Estimated Number of Incident Symptomatic LB Cases in Adults, 2019	Estimated Number of Surveillance-Reported LB Cases in Adults, 2021	Estimated Number of Symptomatic LB Cases in Adults, 2021
672	3975	5.9	1677	9894	1021	6204

**Table 3:** Sensitivity analysis: under-ascertainment multipliers in Lublin and estimated number of symptomatic Lyme borreliosis (LB) cases in Lublin adults in 2021 using 5- and 20-year durations of antibody detection.

Estimated Number of Incident Symptomatic LB Cases in Adults, 2010		Under-Ascertainment Multiplier (Based on 2010 data)		Estimated Number of Surveillance-Reported LB Cases in Adults, 2021	Estimated Number of Incident Symptomatic LB Cases in Adults, 2021	
**5 Years**	20 Years	5 Years	20 Years		5 Years	20 Years
7951	1988	11.8	3.0	1021	12,048	3063

## Data Availability

All data generated or analyzed during this study are included in this published article or are available from the corresponding author on reasonable request.

## Abbreviations

*Borrelia burgdorferi*	*B. burgdorferi*
LB	Lyme borreliosis
ELISA	enzyme-linked immunoassay
